# Evaluating different national strategies to contain the COVID-19 pandemic before mass vaccination

**DOI:** 10.7189/jogh.11.01004

**Published:** 2021-05-15

**Authors:** Igor Rudan

**Affiliations:** Centre for Global Health, the Usher Institute, The University of Edinburgh, Scotland, UK

About a year ago, when the first wave of the pandemic in the Northern Hemisphere was ending, I proposed that the countries of the world would need to choose between four basic strategies in the further course of their respective responses [1]:

- “The complete suppression of the virus”, the implementation of which requires the practical “textbook” application of epidemiological measures: careful border control, two-week quarantine measures for all those who enter the country, a tremendously serious approach to all detected cases, the rapid detection and subsequent isolation of all of the contacts of an infected individual; and, if necessary, a temporary, short-term “lockdown” in limited areas with extensive testing accompanying it. These measures would include the cooperation and discipline of the population, as well as adherence to all of the anti-epidemic measures [1]. With this approach, which isn't particularly financially expensive for most countries, it was possible to spend the vast majority of the time without the fear of the pandemic during 2020 and throughout the course of 2021 to date (this article is relevant to 20 April 2021). In the countries which chose this approach, life carried out more or less as it normally would, resembling the time before the pandemic struck. Examples of such countries are Vietnam, Taiwan, Laos, Thailand and Bhutan, which have recorded less than 1 death from COVID-19 per million inhabitants; followed by China, Cambodia, Singapore, and New Zealand, with less than 10 deaths per million; then, by Japan, Australia, Malaysia, South Korea and Myanmar, with less than 100 deaths per million inhabitants [2]. To date (20 April 2021), these nations have performed the best in protecting their citizens from dying from a new infectious disease. Several of these nations have also shown that countries don't need to be particularly wealthy in order to successfully address this pandemic.
- A “strong first line of defence”, in which a country chooses to introduce less stringent measures which allow for a certain level of coexistence with the virus, but which do not allow the virus to spiral out of control and begin spreading freely among the population. This strategy is based on scientific knowledge and technology, as well as on intensive testing and the isolation of the contacts of the infected. In addition, it also requires the cooperation and discipline of the population. Examples of this strategy are countries that had a very strong capacity to test-and-trace, and in addition, that deployed electronic applications for the tracking of contacts [1]. Some countries from the first group can also be included here, because they also opted to use the methods of this second group, such as the likes of Singapore, South Korea and Taiwan. Along with them, other typical representatives are the United Arab Emirates (UAE), Qatar, Norway, Finland and Iceland, which to date have recorded 100-200 COVID-19 deaths per million inhabitants; followed by Denmark, Oman, Kuwait and Bahrain, with 200-400 deaths per million inhabitants [2].
- “The relaxation and subsequent tightening of measures” was a strategy resorted to by many countries following the first wave of the pandemic. Unlike the first two strategies, which were proactive in their attempt to stay “ahead” of the virus and manage the crisis, this strategy left countries in a passive position towards the virus all too often. Owing to that, the spread of the virus conditioned the stringency of the measures and the way of life of the entire population [1]. This strategy has since proved to be risky. Many nations simply failed to introduce their measures at the time in which they were able to act in a preventative manner, thus warding off the spread of the wave and averting a large number of deaths. Instead, the measures were introduced too late, when the virus would already be spreading freely among the population and the first line of defence would have been breached. Examples of these countries are Canada and Israel with 500-700 COVID-19 deaths per million inhabitants, followed by Greece, Germany, Ireland and the Netherlands which had 800-1000 deaths per million inhabitants. This is also how Austria, Switzerland, Argentina and Chile reacted, which today (20 April 2021) have 1000-1500 deaths per million inhabitants, followed by France, Spain, Italy, Croatia, the United Kingdom, Mexico and Peru, with 1500-2000 deaths per million inhabitants. Finally, several of the hardest-hit countries to date - Belgium, the Czech Republic, Slovakia, Hungary, Bulgaria, Bosnia and Herzegovina, North Macedonia, Montenegro and Slovenia - have recorded more than 2000 deaths per million inhabitants [2].
- “The liberalisation of personal risk management”, which arose as a possible option only when sufficient evidence emerged from seroprevalence studies, ie, the presence of antibodies in a person's blood. These studies confirmed that the death rate among all those who had become infected should not exceed 0.5-1% in the majority of countries [1]. Countries that employed this strategy decided not to pay excessive attention to the pandemic and to preserve their economic activities to the greatest extent possible. Elements of this approach could be found in Sweden (1359 deaths per million inhabitants), the United States (1749 deaths per million) and Brazil (1755 deaths per million), as well as some countries in Africa and the Middle East where the data on deaths from COVID-19 isn't reliable enough [2].

Now, after a year, we can see that all the countries of the world have chosen one of these four paths. What we can conclude from this analysis, when the data are standardised per million inhabitants, is that in some nations  – due to the strategies they selected - COVID-19 has killed hundreds of times more people when compared to some other nations. These differences are really dramatic. More specifically, in Vietnam, Thailand, Bhutan, Taiwan, China, Cambodia, Singapore, and New Zealand, at least 100 times fewer people died from COVID-19 in comparison to many western countries [2]. Most countries in Africa and Central Asia, as well as those in Central America, cannot be fairly included in this analysis, because their official data on the number of deaths can hardly be considered reliable. In addition, African countries have a significantly different age structure than the rest of the world and the proportion of older people on that continent is much lower.

The argument of many western countries that avoided the proactive approach and the introduction of measures was that in this way they would manage to save their economies from the “unimaginable downturns” that would follow the enforcement of stringent anti-epidemic measures and that would surely be in double digits [3].

The International Monetary Fund (IMF) recently published estimates of the change in the Gross Domestic Product (GDP) in all of the countries across the world in the year 2020 [4]. The IMF estimates that the global economy has fallen by 3.3%. This is certainly not a small decline, but it is comparable to some earlier economic crises which came about as a result of other causes. However, this decline does not even remotely reflect the fears from the pre-pandemic period. It is clear that consumer spending has decreased and that sectors such as tourism and hospitality have been hit very hard. Let us now consider whether countries have managed to preserve their economic activity by choosing some strategies to address the coronavirus pandemic over the others?

The percentage change in the gross domestic product in the first group of countries which implemented a strategy of complete suppression of the virus was - from the most successful to the least successful country: Myanmar (GDP change: +3.2%), Taiwan (+3.1%), Vietnam (+2.9%), China (+2.3%), Laos (-0.4%), Bhutan (-0.8%), South Korea (-1.0%), Australia (-2.4%), New Zealand (-3.0%), Cambodia (-3.5%), Japan (-4.8%), Singapore (-5.4%), Malaysia (-5.6%) and Thailand (-6.1%) [4]. It's apparent that as many as seven countries in this group actually experienced economic growth during 2020, or only a minimal decline (up to -1.0%). Australia and New Zealand had a more significant decline, Japan even more so, while for Cambodia, Malaysia and Thailand, large declines were expected due to their dependence on the tourism and transport industries - the latter also being part of the reason why Singapore was significantly affected. In these latter countries, it was inevitable that their economies would experience significant downturns due to their specific structures, despite their excellent response to the COVID-19 crisis in the segment of public health alone.

The percentage change in the GDP in the second group of countries, which relied on their strong first line of defence, was: Norway (-0.8%), Qatar (-2.6%), Finland (-2.9%), Denmark (-3.3%), Cyprus (-5.1%), Bahrain (-5.4%), United Arab Emirates (UAE) (-5.9%), Oman (-6.4%), Iceland (-6.6%) and Kuwait (-8.1%) [4].

Percentage changes in the third group of countries that chose to relax and then tighten their anti-epidemic measures: for countries with less than 1000 COVID-19 deaths per million inhabitants - Ireland (+2.5%), Israel (-2.4%), the Netherlands (-3.8%), Germany (-4.9%), Canada (-5.4%) and Greece (-8.2%). Then, among those nations with 1000-2000 deaths per million inhabitants - Switzerland (-3.0%), Chile (-5.8%), Austria (-6.6%), France (-8.2%), Mexico (-8.2%), Italy (-8.9%), Croatia (-9.0%), the United Kingdom (-9.9%), Argentina (-10.0%), Spain (-11.0%) and Peru (-11.1%). And finally, among those nations with more than 2000 deaths per million inhabitants: Bulgaria (-3.8%), North Macedonia (-4.5%), Hungary (-5.0%), Slovakia (-5.2%), Slovenia (-5.5%), Bosnia and Herzegovina (-5.5%), Czech Republic (-5.6%), Belgium (-6.4%) and Montenegro (-15.2%) [4].

If we look at the percentage changes in GDP in the fourth group, for Sweden it was -2.8%, for the United States -3.5%, and for Brazil -4.1% [4].

When delving deeper into the analysis of these figures, one should be very cautious for at least several reasons. First of all, the official death toll from COVID-19 has been underestimated in almost all the countries of the world, and only a comparison with the average mortality rates of several previous years will give a more accurate picture of the more realistic death toll [5]. On the other hand, the impact of the pandemic on GDP growth or decline had different components. One part of the effect was related to the introduction of varying anti-epidemic measures worldwide. Some countries were simply more exposed to the negative effects of the pandemic events and measures than others. This was related to the structure of their own economies and their dependence on the economies of other nations. Another part had more to do with their own response, internal organisation and self-sufficiency. Only when the careful additional standardisation of all of these effects can be implemented will it be possible to properly determine the success of their responses to the COVID-19 pandemic before the advent of the vaccines.

Still, some general conclusions are possible even from the early data that we now have.

The first general conclusion is that the differences between the success of national strategies in saving human lives were indeed very large. As epidemiologists had expected, the “textbook” epidemiological approach to protecting the population from infectious diseases, as implemented by the first group of countries, has led to a significantly lower number of deaths to date (ranging from 0–100 deaths per million inhabitants). Furthermore, reliance on a strong first line of defence, testing, technological monitoring and the isolation of contacts of the infected typically led to slightly more deaths occurring (in the range of 200–400 deaths per million inhabitants). These two strategies imply a proactive approach, as well as close cooperation between the government and the population in the implementation of the chosen anti-epidemic measures.

Then comes the strategy of “tightening up and easing anti-epidemic measures” based on the monitoring of the number of new cases. This strategy often ended up surprising countries, and resulted in figures from 500 to more than 2000 deaths per million inhabitants, depending on the timeliness and appropriateness of the measures introduced. Finally, we come to a group of countries cited as examples of relying on the caution of the citizens themselves, but which also introduced different preventative measures at various stages of the pandemic. In the United States and Brazil, there were also considerable differences in regional responses, and officially, 1300-1800 COVID-19 deaths per million inhabitants were recorded. However, the nations in this group had periods in which there was a significant possibility that the actual number of deaths due to COVID-19 was greater than reported, during the times when their health systems were under heavy strain. As such, their death rates should also be interpreted with some caution, but this is also true for most other countries which experienced large epidemic waves. The United States' Centre for Disease Control has already indicated that the death toll could be significantly higher during the period of waves of the pandemic hitting the country [6], while the Institute for Health Metrics and Evaluation also believe that the death toll in the USA will need to be revised upwards [7].

Another important conclusion that can be drawn is that in terms of preserving the economy throughout 2020, the countries of the world also differed significantly. A few of them even managed to grow economically –  Myanmar, Taiwan, Vietnam, China and Ireland. In addition, several African countries which produce food and natural resources for other pandemic-affected countries also recorded economic growth. However, these were rare exceptions to the rule in the year 2020. There were only a few countries globally that were well-placed economically in the case of the pandemic, while the vast majority of other countries in the world were challenged quite harshly by that context.

Examples of countries whose economies have been the hardest hit, regardless of their pandemic response strategy, are island nations around the world known mainly for their tourism industries: Seychelles (GDP change: -13.4%), Mauritius (-15.8%), The Bahamas (-16.3%), Barbados (-17.6%) and Maldives (-32.2%). The aforementioned countries are examples where global developments related to the pandemic have had a significantly greater impact on their economies than their own pandemic response could have. In fact, their responses to the public health challenges were generally quite good. Many of these nations have followed the response principles of the countries from the first two groups, so the number of deaths (when expressed per million inhabitants, compared to others, although these countries are smaller) stood at 50-400 per million.

However, there is little doubt that in the course of the pandemic, ie, before the vaccines have a significant impact on the results in the next phase, the best results based on the criteria of saving lives as well as preserving the economy were achieved by the first group - Taiwan (a nation with 24 million inhabitants), Vietnam (96 million), Myanmar (54 million), China (1.4 billion), Laos (7 million), Bhutan (1 million) and South Korea (52 million). All of these countries decided to introduce classic epidemiological measures used to control infectious diseases. They had a notably small number of deaths, and they also managed to achieve economic growth. In the cases where economic growth wasn't achieved, only a very slight decline was recorded.

Therefore, we are able to draw two valuable lessons from the first 16 months of the COVID-19 pandemic. The first one is that active management of the COVID-19 pandemic was very important. It ensured good results in saving human lives. A passive response to the threat of the virus leads to its free spread among the population and a large number of deaths. In comparison, applying classic epidemiological measures with tight border closures, quarantine for passengers arriving in the country and decisive response to each focal point with diligent testing, tracing and isolating contacts is sufficient. To mount this kind of effective response, countries need a functional public health infrastructure to implement the traditional epidemiological measures. We learned that in many western countries this infrastructure was either underdeveloped or neglected early in the pandemic.

Another important lesson we have learned is that the initial dilemma about whether we should work to save human lives or preserve the economy was a false one. It doesn't need to be a choice between one or the other. It has been shown that active implementation of classic epidemiological measures enables an almost normal, pre-pandemic way of living within a country's national borders. It favours the continuation of economic growth and it simultaneously saves human lives. Other strategies will have worse results either for public health, or for the economy, or indeed for both. The figures we have accrued during the first 16 months of the pandemic indicate that much, even when all uncertainty about them is taken into account. If such an epidemiological approach is not feasible for national or local policymakers, the analysis presented in this article allows an assessment of acceptable outcomes and adjustments of strategies accordingly.

The phase of the pandemic in which vaccines were not available is now gradually drawing to a close. In that phase, epidemiological measures to control the spread of this new infectious disease needed to be entirely relied upon and were of paramount importance. In the next phase of the pandemic, the number of deaths and economic growth within individual countries will be affected by entirely different factors. First, it will be important to monitor how quickly will some countries vaccinate the vast majority of their citizens. It will also be of interest how much of the population in certain countries will refuse vaccination. We will need to determine how long will protection provided by the vaccine last. We should also expect further useful drugs for hospital treatment, in addition to the already effective dexamethasone. In addition, one of the dominant topics in the months ahead will be close scrutiny of the properties of new strains and/or variants of the virus, which could spread more rapidly or become more dangerous to human health. The biggest fear is that the new strains could become resistant to our vaccine-induced immunity. Therefore, the scientific community will be focused in 2021 on very careful monitoring and studying of the characteristics of all of the new strains of this virus.
